# Choice of medicine program: A single-institution study

**DOI:** 10.1016/j.amsu.2022.104410

**Published:** 2022-09-02

**Authors:** Nadeem Ikram, Ahmed Hafez Mousa, Asim Muhammad Alshanberi, Salwa Agha Mohammad, Hanin Radwan, Muhammad Awais, Mudassar Majeed

**Affiliations:** a Department of Microbiology, Medicine Program, Batterjee Medical College, Jeddah, 21442, Saudi Arabia; b Medicine Program, Batterjee Medical College, Jeddah, 21442, Saudi Arabia; c Department of Community Medicine and Pilgrims Health Care, Umm Alqura University, Makkah, Saudi Arabia; d Department of Pathology, Shahida Islam Medical and Dental College, Lodhran, Pakistan

## Abstract

**Introduction:**

The decision to apply for medical school is the first and one of the most important career choices that a physician will ever make and the motives for choosing a career in medicine seem to remain relatively stable during medical school. Our study aimed to investigate what motivated the students, their satisfaction with the PBL curriculum and their plans following graduation.

**Methods:**

A cross-sectional descriptive study was carried out from Jan to March 2021 at Batterjee medical college, Jeddah. The data was collected on a questionnaire from undergraduate students of first year till the internship year.

**Results:**

Among the 112 students who completed the questionnaire, 85 (75.9%) chose studying medicine to be their own choice, with service to humanity 56 (50%) being the main reason, followed by monetary 20 (18.8%) reasons. Generally 78 (69.6%) of the responders preferred the PBL curriculum over the traditional one. Most of the students 42 (37.5%) planned to undergo the residency program in Saudi Arabia, with general surgery being the specialty preferred by 33 (29.5%) students.

**Conclusion:**

The findings in our study suggest that altruism is the most common influencing factor that led students joining a medical college

## Highlights

• The decision to apply for medical school is the first and one of the most important career choices that a physician will ever make and the motives for choosing a career in medicine seem to remain relatively stable during medical school.
• This study outlines the factors underlying a student's decision to join a medical school.
• The participants' choice of specialty in case of residency shows significant contrast between the preclinical and clinical years, with surgery and internal medicine being favored by the preclinical students (36.3% and 20.0%, respectively) in contrast to the clinical students (12.5% and 12.5%) respectively.
• The findings in our study suggest that altruism is the most common influencing factor that led students to join a medical college.

## 1 Introduction

There is mushroom growth of schools, both public and private, in every country. The effect of schooling influences the academic performance of students in the medical school. AlMously et al. (2013) reported that English language proficiency was the significant predictor of academic performance in Saudi Medical school [1]. In another study conducted by Costa-Santos et al. (2018), it was found that students from public secondary schools seem to be better prepared for medical school teaching methodologies than their colleagues from private ones [2]. Moreover, a national-based cohort study in the UK by Mwandigha et al. (2018) reported that the undergraduate achievement was inversely related to secondary school level performance [3].

Education in the Kingdom of Saudi Arabia is segregated by gender, with the government providing general education in the form of an equivalent curriculum for both, besides taking an equivalent annual exam. Primary school lasts six years, followed by three years of middle school where student begins studying the English language. High school consists of another three years where students either follow the scientific or the literary track. Students opting to study in a medical school and choosing the scientific track will have their focus directed at biology, chemistry, physics and math. This is followed by a final standardized exam consisting of a General Aptitude Test (GAT) (Qudurat), and Standard Achievement Admission Test (SAAT) (Tahsili) [4]. There is a total of 38 medical colleges in Saudi Arabia, 12 of

which are private and 26 are governmental. Out of these, 7 are in Jeddah, with 4 private and 3 governmental. Deciding to apply for medical school is the first but also one of the most important career choices that a physician will ever make and the motives for choosing a career in medicine seem to remain relatively stable during medical school according to Scott et al. (2012) [5]. Our study aimed to investigate what motivated the students, their satisfaction with the PBL curriculum and their plans following graduation. This study was done in compliance with the STROCSS 2021 criteria [23].

## 2 Methodology

This was a cross-sectional study conducted in Batterjee College, Jeddah over a period of two months from (Jan 1, 2021 to March 1, 2021). The students studying in medicine program were included in the study. Students in the preparatory year were excluded from the study. The data was collected by a questionnaire containing questions pertaining to demographic details, school education, student's choice of medicine and their satisfaction with the college's curriculum and post-graduation plans. Survey was conducted after obtaining consent to participate in the study. The study was carried out after approval of Institutional Review board at Batterjee College RES-2020-0010. The data was analyzed using SPSS (statistical package for social sciences) version 22.

## 3 Results

A total of 112 students participated in our study, including 52 males (46.4%) and 60 females (53.6%), between the ages of 18–26 years old with a mean of 21.8, S. D ± 1.7.

Expat students were more compared to the Saudi students, and majority were unmarried. Students from the first to sixth year of the medicine program were involved in the study (including internship year), with about three-fourths of the participants being from pre-clinical years.

Majority of students completed the primary and secondary education in Saudi Arabia undergoing the Saudi curriculum set by the ministry of education. The details of other curriculums studied are displayed in Table 1.

**Table 1:** Demographic and educational profile of the participants.

Characteristic	Frequency (%)			
Gender				
Male	52 (46.4%)			
Female	60 (53.6%)			
Age	Male (n = 52)	Female (n = 60)		Average
Mean	21.9 S.D. ± 1.7	21.7 S.D. ± 1.7		21.8 S.D. ± 1.7
Max	25	26		26
Min	19	18		18
Nationality				
Saudi	27 (51.9%)	27 (45%)		54 (48.2%)
Non-Saudi	25 (48.1%)	33 (55.0%)		58 (51.8%)
Marital Status				
Single	50 (96.2%)	58 (96.7%)		108 (96.4%)
Married	2 (3.8%)	2 (3.3%)		4 (3.6%)
Year of Study				
Preclinical	34 (65.4%)	46 (76.7%)		80 (71.4%)
Clinical	18 (34.6%)	14 (23.3%)		32 (28.6%)
School Education				
Country	Grades 9 and 10		Grades 11 and 12	
KSA	107 (95.5%)		106 (94.6%)	
Outside KSA	5 (4.5%)		6 (5.4%)	
Curriculum				
Local Saudi	75 (70%)		75 (70%)	
British	10 (8.9%)		9 (8%)	
American	8 (7.1%)		8 (7.1%)	
Other International	19 (16.9%)		20 (17.8%)	

Majority of students 87 (77.7%) joined medical school on their own choice, 17 (15.2%) were influenced by their parents or relatives, and 8 (7.1%) were forced by their parents to study medicine.

The motive behind admission into a medical school was serving the humanity 56 (50%), as shown in Table 2.

**Table 2:** Reasons for selecting medicine program.

Variable	Frequency (%)		
Reason for selection	Male (n = 52)	Female (n = 60)	Total
My own choice	41 (78.8%)	46 (76.7%)	87 (77.7%)
Influenced by parents/relatives	8 (15.4%)	9 (15%)	17 (15.2%)
Forced by parents	3 (5.8%)	5 (8.3%)	8 (7.1%)
Motive			
Serving humanity	17 (32.7%)	39 (65%)	56 (50%)
Earn more money	13 (25%)	8 (13.3%)	21 (18.8%)
Gain respect	8 (15.4%)	2 (3.3%)	10 (8.9%)
Other	14 (26.9%)	11 (18.3%)	25 (22.3%)

In addition, about three-fourths of the participants (73.2%) planned to proceed for residencies following graduation, with 42 (37.5%) students to do residency in Saudi Arabia and 40 (35.7%) in a country outside Saudi Arabia.

Few students 19 (17%) students planned to take the USMLE, among which twice as many are females and from clinical years compared to males and preclinical students. Only 2 male students planned to become general practitioners.

The specialties most frequently chosen by students who plan on a residency were as follows, General Surgery 33 (29.5%), Internal Medicine 20 (17.9%), Pediatrics 16 (14.3%), Ophthalmology 8 (7.1%).

The preclinical students preferred residency in general surgery as compared to students in the clinical years as shown in Table 3.

**Table 3:** Specialty in case of residency and its motive based on gender and clinical stage.

Variable	Frequency (%)				
Plan after graduation	Male (n = 52)	Female (n = 60)	Preclinical (n = 80)	Clinical (n = 32)	Total
Residency KSA	13 (25%)	29 (48.3%)	26 (32.5%)	16 (50%)	42 (37.5%)
Residency outside KSA	26 (50%)	14 (23.3%)	31 (38.8%)	9 (28.1%)	40 (35.7%)
USMLE	6 (11.5%)	13 (21.7%)	16 (20%)	3 (9.4%)	19 (17%)
General practitioner	2 (3.8%)	0	0	2 (6.3%)	2 (1.8%)
Others	5 (9.6%)	4 (6.7%)	7 (8.8%)	2 (6.3%)	9 (8%)
Specialty in case of residency					
General Surgery	14 (26.9%)	19 (31.7%)	29 (36.3%)	4 (12.5%)	33 (29.5%)
Internal Medicine	11 (21.2%)	9 (15%)	16 (20%)	4 (12.5%)	20 (17.9%)
Pediatrics	7 (13.5%)	9 (15%)	10 (12.5%)	6 (18.8%)	16 (14.3%)
Ophthalmology	3 (5.8%)	5 (8.3%)	6 (7.5%)	2 (6.3%)	8 (7.1%)
OB/GYN	1 (1.9%)	2 (3.3%)	2 (2.5%)	1 (3.1%)	3 (2.7%)
Other	16 (30.8%)	16 (26.7%)	17 (21.3%)	15 (46.9%)	32 (28.6%)
Motive					
Personal Interest	41 (78.8%)	54 (90%)	68 (85%)	27 (84.4%)	95 (84.8%)
Earn more money	4 (7.7%)	3 (5%)	6 (7.5%)	1 (3.1%)	7 (6.3%)
Other	7 (13.5%)	3 (5%)	6 (7.5%)	4 (12.5%)	10 (8.9%)

## 4 Discussion

This study outlines the factors underlying a student's decision to join a medical school.

Majority of the study participants' response indicated that selection of medicine for their bachelor's degree in college was their own choice, followed by parental influence. This finding was similar to the study reported by Hassan et al. (2020) where 64.6% joined the medical profession by their own will, 26% were influenced by their parents [6]. Different results were obtained in the study conducted by Zayabalaradjane et al. (2018), where parental influence made up more than 40% of the participant responses [7].

Majority of the students (50%) opted medicine profession to serve humanity, which supports the results obtained from another similar study conducted by Giri et al. (2015) which reported service to the community (58.5%) to be the students' main reason for joining the medical profession [8]. Almost 70% of the students were satisfied and preferred problem based learning as compared to traditional teaching methodology.

Study conducted by Nath et al. (2007), Kuriakose et al. (2015), Harth et al. (1990) and Razali et al. (1996) showed similar results as serving the sick society respectively [[9], [10], [11], [12]]. Monetary reason was the other motive, also reported by Nath et al. (2007) in which 13.8% of the students had chosen the medical profession for monetary reasons [9]. A study conducted in Egypt by Kabil et al. (2018) also ranked prestige as the most highly considered motivational factor followed by financial security [6,13]. Twice as many females in comparison to males chose “to serve humanity” and almost half as many (13.3%) of the females chose “to earn more money”. Choosing medicine program becasue of prestige was opted by 10 (8.9%) students. Findings in the study by Kabil et al. (2018) support our findings, where it was reported that males are more inclined towards prestige and high income in relation to females [13]. Kuriakose et al. (2015) and Hassan et al. (2020) did not support differences between the two genders [6,10].

There were few students 8 (7.1%) who never wanted to become doctors but were forced to pursue a medical career by their parents. These students usually do not continue medicine profession after graduation, and they should be particularly followed, and academic counseling be offered for planning their future. Firdous et al. (2019) concluded that this category of students generally shows low levels of life satisfaction and academic achievement, emphasizing on the importance of guidance and support by their institutions [14].

Generally, Gulf country citizens prefer residency program in their respective countries or be sponsored for a residency program abroad [15]. In this study, we found that most students want to proceed for a residency within Saudi Arabia.

General surgery was the preferred specialty, followed by internal medicine, similar to study carried by Mohammed et al. (2020) [16]. Meanwhile, Alshahrani et al. (2014) reported internal medicine to be the top specialty of choice [17]. Almost 31.7% of female students in our study have cited surgery as their specialty of choice while only 3.3% have chosen Obstetrics and Gynecology. A study by Ismail and Kevelighan (2019) support our finding, where only 3.9% of the females considered Obstetrics and Gynecology as their first-choice specialty [18]. Another study by Mahha et al. (2020) reported 8.8% of the students consider the specialty as their first choice [19]. Elspeth et al. (2014) suggests that females are significantly more likely to pursue general surgery if they train in a hospital with abundant and prominent female role models [20].

The participants' choice of specialty in case of residency shows significant contrast between the preclinical and clinical years, with surgery and internal medicine being favored by the preclinical students (36.3% and 20.0%, respectively) in contrast to the clinical students (12.5% and 12.5%) respectively. According to Vo et al. (2017), this shift in interest between preclinical and clinical students could be explained by the clinical students' present focus on specialties that allow greater flexibility and control over work hours following their exposure to real-life circumstances during their hospital training [21].

Pediatrics was the most frequently chosen specialty by clinical students. Nieuf et al. (2005) reported that preclinical students are not familiar with the differences between specialties when compared to those with clinical experience, hence their perception is mostly dominated by the social aspects of the profession such as prestige and income which they associate with surgeons, rather than patient contacts [22]. Few students (1.8%) planed for general practice after graduation.

## 5 Limitations and recommendations

The fact that our study was conducted on a single institution reflects a limitation to our study. We suggest that further studies in the future get done based on comparing outcomes from different institutions.

## 6 Conclusion

The findings in our study suggest that altruism is the most common influencing factor that led students to join a medical college. Most students plan to proceed for a residency. However, currently the target specialty does not seem to be a solid option with trends showing a decreasing tendency for surgery moving from the preclinical to clinical years. Few students opted to become general practitioners.

## Ethical approval

Ethical approval has been given by the Institutional Review Board (IRB) of our institution, Batterjee Medical College, Jeddah, Saudi Arabia.

## Sources of funding

No Funding For This Research.

## Author contributions

Drafting of the manuscript: Ahmed Hafez Mousa, Asim Muhammad Alshanberi, Nadeem Ikram, Salwa Agha Mohammad, Hanin Radwan, Muhammad Awais, Mudassar Majeed.

Critical revision of the manuscript for important intellectual content:

Ahmed Hafez Mousa, Asim Muhammad Alshanberi, Nadeem Ikram, Salwa Agha Mohammad, Hanin Radwan, Muhammad Awais, Mudassar Majeed.

## Registration of research studies

1. Name of the registry:

2. Unique Identifying number or registration ID:

3. Hyperlink to your specific registration (must be publicly accessible and will be checked):

## Guarantor

Ahmed Hafez Mousa, corresponding author of the manuscript, accept full responsibility for the work and the conduct of the study, had access to the data, and controlled the decision to publish.

## Consent

The study was carried out after approval of Institutional Review board at Batterjee Medical College RES-2020-0010. Informed verbal and written consent were taken from the students.

## Declaration of competing interest

No Conflicts Of Interest
